# Increased Systemic Immune-Inflammation Index Predicts Disease Severity and Functional Outcome in Acute Ischemic Stroke Patients

**DOI:** 10.1097/NRL.0000000000000464

**Published:** 2022-09-17

**Authors:** Lu Huang

**Affiliations:** Department of Health Management Center, The First Affiliated Hospital of Chongqing Medical University, Chongqing, China

**Keywords:** acute ischemic stroke, systemic immune-inflammation index (SII), system inflammation response index (SIRI), severity of stroke, outcome

## Abstract

Systemic immune-inflammation index (SII) and system inflammation response index (SIRI) have been recently investigated as novel inflammatory and prognostic markers. Our study aimed to investigate the relationship between SII and SIRI index and severity of stroke, and to analyze the prognostic value in acute ischemic stroke (AIS) patients.

**Methods:**

The SII is defined as platelet×(neutrophil count/lymphocyte count), SIRI is defined as neutrophil count×(monocyte count/lymphocyte count). We plotted receiver operating characteristic curves of SII and SIRI for poor outcomes and calculated area under the curve (AUC) values and cutoff values. Multivariate logistic regression analysis was performed to analyze the association between SII/SIRI index and poor functional outcome.

**Results:**

We included 234 AIS patients [mean age 69 (57-78) years; 50.4% male]. Both SII and SIRI were higher in the moderate-to-severe stroke group than in the mild stroke group [932.73 (569.84-1610.90) vs. 581.21 (386.98-1015.59), *P*<0.001 and 2.00 (1.24-3.13) vs. 1.35 (0.83-1.92), *P* <0.001]. The area under the receiver operating characteristic curve (area under the curve) value of SII (0.678, 0.608-0.748, *P*<0.001) tested a similar discriminatory ability compared with SIRI (0.682, 95% CI (0.612-0.751), *P*<0.001). Multivariate logistic regression analyses showed that SII was significantly associated with poor prognosis at discharge of AIS patients [adjusted odds ratio (95% confidence interval): 2.350 (1.149-4.803), *P*=0.019)], conversely, SIRI had no prognostic value.

**Conclusions:**

Higher SII and SIRI indexes were correlated with greater risk of stroke severity, meanwhile SII could be useful for predicting adverse clinical outcomes after AIS.

Globally, stroke is the second leading cause of death in persons older than 60 years.^1^ In China, the incidence of cerebrovascular disease has exceeded that of heart disease, becoming the first leading cause of death and disability in adults.^2^,^3^ At present, the number of stroke patients is 13 million, with more than 2 million new patients each year and prevalence is increasing.^4^ Acute ischemic stroke (AIS) is the most common type of stroke, comprising 80% of all types of stroke, resulting in a huge burden on the society. Therefore, it is significant to explore rapidly effective measurable biomarkers to predict disease development and functional prognosis which may further improve the recovery of patients.

Immune cells and inflammation play important roles in the pathogenesis of ischemic stroke.^5^ Cell counting and their combinations, such as neutrophil to lymphocyte ratio (NLR) and platelet-to-lymphocyte ratio (PLR), have been regard as value of prognosis in AIS.^6^–^8^ Those indexes, derived from peripheral blood tests are easily available, and are considered to be classic hematological markers of systemic inflammation. Recently, a growing body of evidence suggests that systemic immune-inflammation index (SII) and system inflammation response index (SIRI) have been regarded to be related to poor outcomes of cancers, such as gallbladder cancer and colorectal cancer.^9^–^11^ Thus, we propose a hypothesis that the levels of SII and SIRI could be associated with stroke severity and early functional outcomes at discharge of AIS patients.

## MATERIALS AND METHODS

### Study Population

A cross-sectional study was conducted from October 2017 to May 2019, all patients with AIS were consecutively included in the study and collected from the Department of Neurology of the First affiliated Hospital of Chongqing Medical University. Patients were included if they met the following criteria: (1) aged 18 years or older; (2) admission for first-ever AIS within 24 h; The exclusion criteria were as follows: (1) infection within two weeks before stroke; (2) patients with malignant tumor and autoimmune diseases; (3) history of transient ischemic attack, cerebral infarction, intracranial hemorrhage, aneurysmal subarachnoid hemorrhage, and venous sinus thrombosis; (4) severe hepatic and/or renal insufficiency. AIS was defined according to World Health Organization recommendations (defined stroke as a “neurological deficit of cerebrovascular cause that persists beyond 24 hours or is interrupted by death within 24 hours”).^12^ The clinical diagnosis of stroke was confirmed by computed tomography scans or diffusion weight imaging after admission.

**FIGURE 1:**
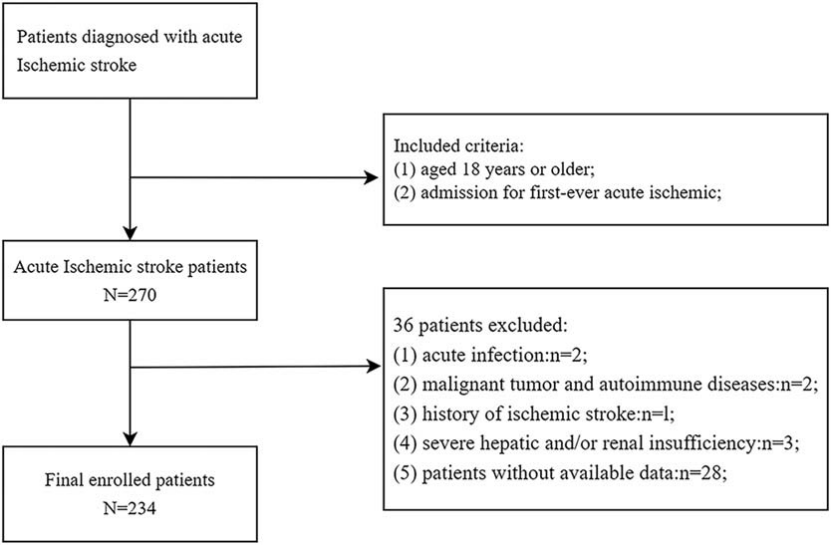
Flow chart.

Among the enrolled patients, 270 were diagnosed with AIS, and there in 234 patients were involved in our analysis after excluding those with infection within 2 weeks before admission (n=2); malignant tumor and autoimmune diseases (n=2); history of ischemic stroke(n=1); severe hepatic and/or renal insufficiency (n=3); and patients without available complete blood count (n=28) (Fig. 1).

The study was approved by the Ethics Committee of the First Affiliated Hospital of Chongqing Medical University. All procedures were carried out in accordance with the code of ethics of the 1975 Declaration of Helsinki.

### Study Protocol and Data Collection

#### Clinical Stroke Severity and Outcomes Measures

The National Institutes of Health Stroke Scale (NIHSS) was used to evaluate the stroke severity within 24 hours after admission, which ranged from 0 to 42 and the higher the score, the more severe the disease was. Mild stroke was defined as NIHSS scores≤5, moderate-to-severe stroke (NIHSS score >5) which was recommended in other study.^13^ Stroke subtype was classified according to the the Trial of Org 10172 in Acute Stroke Treatment (TOAST) criteria. The functional outcomes were assessed by the modified Rankin scale (mRS) at discharge (favorable outcome, mRS score ≤2; poor outcomes, scores >2).^14^

#### Clinical Data Collection

The data collected were obtained from medical records. At baseline, demographic data (age and sex), history of vascular risk factors (including hypertension, diabetes mellitus, atrial fibrillation, smoking, and drinking), systolic blood pressure (SBP), diastolic blood pressure (DBP), treatment after admission (antiplatelet, anticoagulation, thrombolysis therapy) were obtained. Fasting blood samples were collected from the cubital vein from each patient in the early morning within 24 hours of admission. Hematological markers, including white blood count (W), neutrophil count (N), lymphocyte count (L), platelet (P), monocyte count (M), and high-sensitivity C-reactive protein (Hs-CRP) were assessed. These counts were used to calculate the following inflammatory biomarkers: neutrophil to lymphocyte ratio (NLR=N/L), platelet to lymphocyte ratio (PLR=*P*/L), and systemic immune-inflammation index (SII=P×[N/L]), and system inflammation response index (SIRI=N×[M/L]).

### Statistical Analysis

Statistical analysis was performed using SPSS 22.0 software. Normally distributed data were expressed as mean±SD, and nonnormally distributed data as medians and interquartile range. Comparison between group was performed by Mann-Whitney *U* test, chi-square test where appropriate. A nonparametric Kruskal-Wallis test was used to compare group differences for nominal variables. Correlation analysis used Spearman correlation analysis. Receiver operating characteristic (ROC) analysis was performed to identity the optimal cut-off values of SII and SIRI indexes for discrimination of clinical outcome after stroke. The variables with statistical significance of *P*<0.1 in the univariate analysis were included in multivariate logistic regression analysis for screening independent risk factors of unfavorable outcomes. *P* value <0.05 were considered as statistically significant.

## RESULTS

### Patient Characteristics

A total of 234 AIS patients (118 men and 116 women) with a mean age of 69 (57-78) years were included in the study. Demographic features and risk factors are shown in Table 1. The mean SBP was 158.4±24.5 mmHg and diastolic BP was 90.4±15.1 mmHg. Vascular risk factors included hypertension (n=164, 70.1%), diabetes mellitus (n=53, 22.6%), atrial fibrillation (n=28, 12.0%), Smoking (n=80, 35.9%), and drinking (n=72, 30.8%). Patients in moderate-to-severe stroke group higher SBP (mmHg) (159.5±26.5 vs. 157.6±22.7, *P*=0.031). Compared with the patients in the mild group, the patients in the moderate-to-severe stroke group possessed a significantly higher white blood cell level (7.96 [6.21-10.14] vs. 6.93 [5.65–8.49], *P*=0.002), a higher neutrophil count level (5.76 [4.36–8.23] vs. 4.59 [3.59–6.01], *P*<0.001), and a lower lymphocyte count level (1.19 [0.79–1.61] vs. 1.59 [1.11–1.96], *P*<0.001), higher NLR (5.37 [3.04–8.72] vs. 3.02 [2.11–4.92], *P*<0.001), higher PLR (160.42 [115.02–225.80] vs. 127.67 [98.27–187.53], *P*=0.006). The moderate-to-severe stroke group had a statistically significant difference in higher initial NIHSS score (10(7–16) vs. 3(1–4), *P*<0.001), more patients received thrombolysis (13.3% vs. 5.4%, *P*=0.035) treatment compared with those in the mild stroke group, and a worse functional outcome at discharge with mRS (1(1–3) vs. 3(2–5), *P*<0.001) being higher.

**TABLE 1:** Baseline Characteristics of Patients Stratified by Mild Stroke and Moderate-to-Severe Stroke

Variables	All patients (n=234)	Mild stroke (NIHSS≤5) n=129	Moderate-to-severe stroke (NIHSS>5) n=105	*P*
Age (y)	69 (57-78)	67 (56-78)	70 (62-79)	0.276
Sex ((n, %))	234	129	105	0.422
Man (n, %)	118 (50.4)	62 (48.1)	56 (53.3)	—
Female (n, %)	116 (49.6)	67 (51.9)	49 (46.7)	—
Vascular risk factors				
Hypertension (n, %)	164 (70.1)	91 (70.5)	73 (69.5)	0.866
Diabetes (n, %)	53 (22.6)	23 (17.8)	30 (28.6)	0.051
Atrial fibrillation (n, %)	28 (12.0)	10 (7.8)	18 (17.1)	0.028
Smoking (n, %)	84 (35.9)	49 (38.0)	35 (33.3)	0.461
Drinking (n, %)	72 (30.8)	39 (30.2)	33 (31.4)	0.844
Clinical features				
SBP (mmHg)	158.4±24.5	157.6±22.7	159.5±26.5	**0.031**
DBP (mmHg)	90.4±15.1	92.3±15.3	88.1±14.6	0.673
WBC (10^9^/L)	7.26 (5.84-8.88)	6.93 (5.65-8.49)	7.96 (6.21-10.14)	**0.002**
N(10^9^/L)	5.14 (3.79-7.15)	4.59 (3.59-6.01)	5.76 (4.36-8.23)	**<0.001**
L(10^9^/L)	1.41 (0.94-1.77)	1.59 (1.11-1.96)	1.19 (0.79-1.61)	**<0.001**
P(10^9^/L)	195 (159-234)	198 (162-245)	187 (158-229)	0.411
NLR	3.82 (2.36-5.90)	3.02 (2.11-4.92)	5.37 (3.04-8.72)	**<0.001**
PLR	137.97 (106.12-208.25)	127.67 (98.27-187.53)	160.42 (115.02-225.80)	**0.006**
Hs-CRP (mg/L)	1.85 (0.87-4.26)	1.50 (0.69-3.45)	2.43 (1.09-5.74)	**<0.001**
SII (10^9^/L)	726.28 (450.41-1207.31)	581.21 (386.98-1015.59)	932.73 (569.84-1610.90)	**<0.001**
SIRI (10^9^/L)	1.62 (0.98-2.45)	1.35 (0.83-1.92)	2.00 (1.24-3.13)	**<0.001**
NIHSS score at admission	5 (3-9)	3 (1-4)	10 (7-16)	**<0.001**
Treatment in hospital (n, %)				
Antiplatelet, n (%)	230 (98.7)	128 (99.2)	102 (97.1)	0.222
Anticoagulation, n (%)	27 (11.5)	12 (9.3)	15 (14.3)	0.235
Thrombolysis, n (%)	21 (9.0)	7 (5.4)	14 (13.3)	**0.035**
Short-term outcome				
mRS at discharge	2 (1-4)	1 (1-3)	3 (2-5)	**<0.001**
Poor outcome (n, %)	97 (41.5)	37 (28.7)	60 (57.1)	—
Favorable outcome (n, %)	137 (58.5)	92 (71.3)	45 (42.9)	—
Stroke subtype (n, %)				**<0.001**
Large-vessel occlusive	77 (32.9)	32 (24.8)	45 (42.9)	—
Small-vessel occlusive	70 (29.9)	55 (42.6)	15 (14.5)	—
Cardioembolic	27 (11.5)	8 (6.2)	19 (18.1)	—
Undetermined/unclassified	60 (25.6)	34 (26.4)	26 (24.8)	—

Bold values indicate statistically significant *P*<0.005.

DBP indicates diastolic blood pressure; Hs-CRP, high-sensitivity C-reactive protein; L, lymphocyte count; N, neutrophil count; NIHSS, National Institute of Health stroke scale; NLR, neutrophil to lymphocyte ratio; PLR, platelet to lymphocyte ratio; SBP, systolic blood pressure; SII, systemic immune-inflammation index; SIRI, system inflammation response index; WBC, white blood count.

### SII and SIRI in Relation to Stroke Severity

**FIGURE 2:**
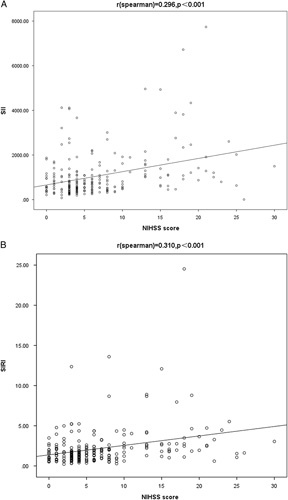
(A) Correlation between SII and NIHSS score; *r*[spearman]=0.296, *P*<0.001. (B) Correlation between SIRI and NIHSS score; *r*[spearman]=−0.310, *P*≤0.001. SII indicates systemic immune-inflammation index; SIRI, system inflammation response index.

Both SIRI and SII were significantly higher in moderate-to-severe group than in the mild stroke group [932.73 (569.84-1610.90) vs. 581.21 (386.98-1015.59), *P* <0.001 and 2.00 (1.24-3.13) vs. 1.35 (0.83-1.92), *P* <0.001]. In addition, the levels of SII and SIRI showed a positive correlation with NIHSS score (*r*=0.296, 0.310, *P*<0.001), as shown in Figure 2.

### SII and SIRI in Relation to Functional Outcome at Admission

**FIGURE 3:**
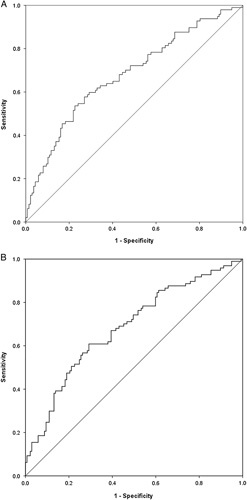
ROC curve of SII and SIRI in patients with AIS. (A) The optimal cut-off values of SII was 1008.33×10^9^/L (AUC=0.678, 95% CI=0.608–0.748, *p*<0.001); (B) The optimal cutoff value of SIRI was 1.79×10^9^/L (AUC=0.682, 95% CI=0.612–0.751, *p*<0.001). AUC indicates area under the curve; CI, confidence interval; SII, systemic immune-inflammation index; SIRI, system inflammation response index.

According to the ROC analysis (Fig. 3), the optimal cut-off values of SII and SIRI were 1008.33×10^9^/L [area under the curve (AUC)=0.678, 95% confidence interval (CI)=0.608-0.748, *P*<0.001] and 1.79×10^9^/L (AUC=0.682, 95% CI=0.612-0.751, *P*<0.001), respectively.

In univariate analysis, SII, SIRI, hs-CRP and NIHSS scores were found to be associated with unfavorable functional outcome at admission. Multivariable logistic regression analysis showed that SII (≥1008.33×10^9^/L) (OR=2.350, 95% CI=1.149-4.803, *P*=0.019) was an important predictor of early functional outcomes at discharge in AIS patients, as shown in Table 2.

**TABLE 2:** Univariate and Multivariate Logistic Regression Analyses Showing the Independent Predictors of Mortality at Discharge

	Univariate			Multivariate		
Variables	OR	95% CI	*P*	OR	95% CI	*P*
Age (y)	1.013	0.991-1.035	0.242	—	—	—
Sex	1.144	0.680-1.926	0.611	—	—	—
SII (≥1008.3×10^9^/L)	3.951	2.245-6.953	**<0.001**	2.350	1.149-4.803	**0.019**
SIRI (≥1.79×10^9^/L)	3.765	2.174-6.522	**<0.001**	1.451	0.713-2.954	0.305
Hs-CRP (≥1.55 mg/L)	3.966	2.237-7.031	**<0.001**	3.149	1.669-5.940	**<0.001**
NIHSS (≥7.5)	5.273	2.896-9.602	**<0.001**	3.580	1.853-6.917	**<0.001**

*P*<0.05 in bold print.

Hs-CRP indicates high-sensitivity C-reactive protein; NIHSS, National Institute of Health stroke scale; SII, systemic immune-inflammation index; SIRI, system inflammation response index.

## DISCUSSION

In the present study, we evaluated the relation between SII and SIRI and stroke severity and prognosis. We found that statistically significant differences in SII and SIRI among subgroups as defined by NIHSS scores. Also, SII was the significant predictor of early functional outcomes at discharge in patients with AIS.

Compared with NLR and PLR, the SII and SIRI indexes might be more reasonable and effective to reflect the overall status of the immune systems of stroke patients. In our study, we used the combination index and have confirmed that SII and SIRI were all associated with stroke severity, meanwhile, SII could be a superior predictor of unfavorable functional outcome, and the risk of poor outcomes at discharge increased 2.350-fold when SII≥1008.33.

Neuroinflammation has drawn increasing attention in recent years. Acute cerebral ischemic injury produces intravascular inflammatory and peripheral immune response. Many studies have emphasized that the inflammatory cascade is activated immediately after vessel occlusion has occurred, resulting in increased brain injury and neurological dysfunction.^5^,^15^,^16^ The SII index includes peripheral lymphocytes, neutrophils and platelets. Thus, SII may be effective to reflect three pathways of thrombus formation, inflammatory response, and adaptive immune response, which are important mechanisms for the development of stroke and may be underlying biomarker for prognosis.^17^ An elevated SII value represents both pro-thrombotic (higher platelet counts) and immune dysregulation (higher neutrophil counts and lower lymphocyte counts) states.^18^

Why is higher SII level related to greater risk of stroke severity and unfavorite outcomes? We hypothesize here several reasons why the SII value is elevated in severe stroke patients. The following mechanisms should be emphasized. Firstly, in the white blood cell family circulating in the periphery, neutrophils are the earliest to infiltrate the lesion within a few hours after stroke, which further leads to the release of inflammatory mediators and directly leads to cell necrosis and apoptosis in the ischemic area.^19^,^20^ Neutrophils are an important source of cytokines, free radicals, and matrix metalloproteinase-9, which induce apoptosis of nerve cells and damage the blood-brain barrier through direct damage to brain tissue.^21^ The destruction of blood-brain barrier not only makes it permeable to white blood cells, but also is associated with various complications of stroke, such as pathologic brain edema, bleeding transformation, and deterioration of neurological function.^22^ Therefore, the increase of neutrophils is an important mediator of ischemic brain injury. Although the role of lymphocytes in ischemic brain injury is controversial, more experimental evidence suggests that some specific subtypes of lymphocytes, particularly CD4+, CD8+ T cells, can produce several cytotoxic substances and pro-inflammatory cytokines. However, some research report that lymphocytes play a key role in inflammation-induced neuroprotection and are the main brain protective immune modulators after AIS.^23^ Secondly, platelets promote brain injury following ischemic stroke.^24^–^26^ In a mice experiment, cerebral ischemia-reperfusion induces platelet necrosis, which in turn modulates injurious neutrophil recruitment and platelet-neutrophil aggregates formation in the brain, impairing cerebral blood flow.^25^ When inflammation is activated, platelets aggregate with circulating leukocytes via direct receptor–ligand interactions, activating platelet function and changing the characteristics of endothelial cells.^27^ A high SIRI corresponds to high neutrophil/monocyte and (or) low lymphocyte counts, which reflects strong pro-inflammatory response mediated by monocytes and neutrophils and lymphocyte-mediated anti-inflammatory response.^28^,^29^ However, more studies are warranted to validate these assumptions.

Up to now, several studies have reported the value of SII and/or SIRI in cerebrovascular diseases. In a 10-year follow-up study of 85,154 individuals, it was showed that elevated SII and SIRI increased the risk of stroke and all-cause death.^30^ Shen’s study discovered SII was a potential predictor in the poor prognosis of patients with acute/subacute cerebral venous sinus thrombosis, especially in male and pregnancy/puerperium female.^31^ Hou et al^13^ found that SII was independently associated with stroke severity. The finding of our research has provided new support for the role of SII/SIRI in AIS patients.

We identified the association of SII and early functional outcomes at discharge with AIS. The ROC curve was used to analyze the predictive value of SII and for the early functional outcome of AIS patients. Results show that SII equal to 1008.33×10^9^/L and SIRI equal to 1.79×10^9^/L are the best truncation value, which help to identify AIS individuals who are at high risk of poor outcome. Further studies are required to validate the findings and identify the underlying mechanisms.

Hs-CRP plays an important role in the inflammatory response and may enhance brain injury by promoting atherosclerosis or lesion plaques.^32^ These results of our study indicate that the plasma hs-CRP levels at admission in the AIS patients are associated with severity of stroke. Compared with moderate-to-severe stroke, patients with mild stroke have lower hs-CRP levels [2.43 (1.09-5.74) vs. 1.50 (0.69-3.45, *P*<0.001], and associated with poor functional outcome (OR=3.149, 95% CI=1.669-5.940, *P*<0.001). During the acute phase of ischemic stroke, hs-CRP in the blood is elevated due to inflammation caused by ischemic brain injury. In uninfected patients, hs-CRP concentration is elevated may reflect the level of neuroinflammatory response after stroke, the higher the hs-CRP level, the more severe the inflammatory response.^33^ Hs-CRP interacts with vascular endothelial cells and other cells, accelerates the process of vascular inflammation, and makes atherosclerotic plaques unstable or even ruptured, leading to a series of pathologic changes and physiological processes, such as leukocyte adhesion, platelet activation and oxidation, and thrombosis.^34^ High plasma hs-CRP levels may present a useful biomarker to identify stroke patients with severe stroke.

There are some limitations to our study. First, this study was a retrospective study conducted in a single center, therefore, there was a selection bias. Second, the sample size is small and should be verified in other larger populations. Third, we have only analyzed the SII and SIRI indices on admission, the dynamic changes should be measured. A multicenter, prospective study will be considered in the future to further explore the relationship between.

## CONCLUSIONS

In summary, severe stroke patients had higher SII and SIRI indexes. Also, SII was an important predictor of early functional outcomes at discharge in AIS patients. Further studies are needed for dynamic monitoring of SII and SIRI indexes to better understand the value of the markers in larger cohorts. Thus, AIS patients with elevated SII and/or SIRI levels should be closely monitored, which may be a potential therapeutic strategy to limit brain damage following ischemic stroke.

## References

[1] Sun H Zou X Liu L . Epidemiological factors of stroke: a survey of the current status in china. J Stroke. 2013;15:109–114. 24324946 10.5853/jos.2013.15.2.109 PMC3779665

[2] Claiborne Johnston S Mendis S Mathers CD . Global variation in stroke burden and mortality: estimates from monitoring, surveillance, and modelling. Lancet Neurol. 2009;8:345–354. 19233730 10.1016/S1474-4422(09)70023-7

[3] Zhao D Liu J Wang M . Epidemiology of cardiovascular disease in China: current features and implications. Nat Rev Cardiol. 2018;16:203–212. 10.1038/s41569-018-0119-4 30467329

[4] Wu S Wu B Liu M . Stroke in China: advances and challenges in epidemiology, prevention, and management. Lancet Neurol. 2019;18:394–405. 30878104 10.1016/S1474-4422(18)30500-3

[5] Anrather J Iadecola C . Inflammation and stroke: an overview. Neurotherapeutics. 2016;13:661–70. 27730544 10.1007/s13311-016-0483-x PMC5081118

[6] Chen C Gu L Chen L . Neutrophil-to-lymphocyte ratio and platelet-to-lymphocyte ratio as potential predictors of prognosis in acute ischemic stroke. Front Neurol. 2020;11:525621. 33569032 10.3389/fneur.2020.525621 PMC7868420

[7] Kakhki RD Dehghanei M ArefNezhad R . The predicting role of neutrophil- lymphocyte ratio in patients with acute ischemic and hemorrhagic stroke. J Stroke Cerebrovasc Dis. 2020;29:105233. 33066938 10.1016/j.jstrokecerebrovasdis.2020.105233

[8] Sun G Yang Y Chen Z . Neutrophil to lymphocyte ratio predicts outcome of stroke by cervicocranial arterial dissection. Front Med. 2020;7:598055. 10.3389/fmed.2020.598055 PMC7729127 33330561

[9] Sun L Jin Y Hu W . The impacts of systemic immune-inflammation index on clinical outcomes in gallbladder carcinoma. Front Oncol. 2020;10:554521. 33194617 10.3389/fonc.2020.554521 PMC7645045

[10] Dong M Shi Y Yang J . Prognostic and clinicopathological significance of systemic immune-inflammation index in colorectal cancer: a meta-analysis. Ther Adv Med Oncol. 2020;12:1758835920937425. 32699557 10.1177/1758835920937425 PMC7357045

[11] Ji Y Wang H . Prognostic prediction of systemic immune-inflammation index for patients with gynecological and breast cancers: a meta-analysis. World J Surg Oncol. 2020;18:197. 32767977 10.1186/s12957-020-01974-w PMC7414550

[12] Stroke--1989. Recommendations on stroke prevention, diagnosis, and therapy. Report of the WHO Task Force on Stroke and other Cerebrovascular Disorders. Stroke. 1989;20:1407–1431. 2799873 10.1161/01.str.20.10.1407

[13] Hou D Wang C Luo Y . Systemic immune-inflammation index (SII) but not platelet-albumin-bilirubin (PALBI) grade is associated with severity of acute ischemic stroke (AIS). Int J Neurosci. 2020;131:1203–1208. 32546038 10.1080/00207454.2020.1784166

[14] Tu WJ Dong X Zhao SJ . Prognostic value of plasma neuroendocrine biomarkers in patients with acute ischaemic stroke. J Neuroendocrinol. 2013;25:771–778. 23701638 10.1111/jne.12052

[15] Vidale S Consoli A Arnaboldi M . Postischemic inflammation in acute stroke. J Clin Neurol. 2017;13:1–9. 28079313 10.3988/jcn.2017.13.1.1 PMC5242162

[16] Maida CD Norrito RL Daidone M . Neuroinflammatory mechanisms in ischemic stroke: focus on cardioembolic stroke, background, and therapeutic approaches. Int J Mol Sci. 2020;21:6454. 32899616 10.3390/ijms21186454 PMC7555650

[17] Xu M Chen R Liu L . Systemic immune-inflammation index and incident cardiovascular diseases among middle-aged and elderly Chinese adults: The Dongfeng-Tongji cohort study. Atherosclerosis. 2021;323:20–29. 33773161 10.1016/j.atherosclerosis.2021.02.012

[18] Chen L Pandey S Shen R . Increased systemic immune-inflammation index is associated with delayed cerebral ischemia in aneurysmal subarachnoid hemorrhage patients. Front Neurol. 2021;12:745175. 34707561 10.3389/fneur.2021.745175 PMC8542972

[19] Darbousset R Thomas GM Mezouar S . Tissue factor-positive neutrophils bind to injured endothelial wall and initiate thrombus formation. Blood. 2012;120:2133–2143. 22837532 10.1182/blood-2012-06-437772

[20] Jin R Yang G Li G . Inflammatory mechanisms in ischemic stroke: role of inflammatory cells. J Leukoc Biol. 2010;87:779–789. 20130219 10.1189/jlb.1109766 PMC2858674

[21] Chamorro Á Dirnagl U Urra X . Neuroprotection in acute stroke: targeting excitotoxicity, oxidative and nitrosative stress, and inflammation. Lancet Neurol. 2016;15:869–881. 27180033 10.1016/S1474-4422(16)00114-9

[22] Petty MA Lo EH . Junctional complexes of the blood–brain barrier: permeability changes in neuroinflammation. Prog Neurobiol. 2006;68:311–23. 10.1016/s0301-0082(02)00128-4 12531232

[23] Liesz A Suri-Payer E Veltkamp C . Regulatory T cells are key cerebroprotective immunomodulators in acute experimental stroke. Nat Med. 2009;15:192–199. 19169263 10.1038/nm.1927

[24] Nording HM Seizer P Langer HF . Platelets in inflammation and atherogenesis. Front Immunol. 2015;6:98. 25798138 10.3389/fimmu.2015.00098 PMC4351644

[25] Denorme F Manne BK Portier I . Platelet necrosis mediates ischemic stroke outcome in mice. Blood. 2020;135:429–440. 31800959 10.1182/blood.2019002124 PMC7005363

[26] Arevalo-Lorido JC Carretero-Gomez J Alvarez-Oliva A . Mean platelet volume in acute phase of ischemic stroke, as predictor of mortality and functional outcome after 1 year. J Stroke Cerebrovasc Dis. 2013;22:297–303. 22005035 10.1016/j.jstrokecerebrovasdis.2011.09.009

[27] Gawaz M Langer H May AE . Platelets in inflammation and atherogenesis. J Clin Invest. 2005;115:3378–3384. 16322783 10.1172/JCI27196 PMC1297269

[28] Jin Z Hao D Song Y . Systemic inflammatory response index as an independent risk factor for ischemic stroke in patients with rheumatoid arthritis: a retrospective study based on propensity score matching. Clin Rheumatol. 2021;40:3919–3927. 33966169 10.1007/s10067-021-05762-z

[29] Yi HJ Sung JH Lee DH . Systemic inflammation response index and systemic immune-inflammation index are associated with clinical outcomes in patients treated with mechanical thrombectomy for large artery occlusion. World Neurosurg. 2021;153:e282–e9. 34217857 10.1016/j.wneu.2021.06.113

[30] Jin Z Wu Q Chen S . The associations of two novel inflammation indexes, SII and SIRI with the risks for cardiovascular diseases and all-cause mortality: a ten-year follow-up study in 85,154 individuals. J Inflamm Res. 2021;14:131–40. 33500649 10.2147/JIR.S283835 PMC7822090

[31] Li S Liu K Gao Y . Prognostic value of systemic immune– inflammation index in acute/subacute patients with cerebral venous sinus thrombosis. Stroke Vasc Neurol. 2020;5:368–373. 32586971 10.1136/svn-2020-000362 PMC7804059

[32] Cai Z He W Zhuang FJ . The role of high high-sensitivity C-reactive protein levels at admission on poor prognosis after acute ischemic stroke. Int J Neurosci. 2019;129:423–429. 30332913 10.1080/00207454.2018.1538139

[33] Yu B Yang P Xu X . C-reactive protein for predicting all-cause mortality in patients with acute ischemic stroke: a meta-analysis. Biosci Rep. 2019;39:BSR20181135. 30718369 10.1042/BSR20181135 PMC6379508

[34] Wei P Han B Zhang WJ . Effect of ticagrelor on the serum level of hs-CRP, ESM-1 and short-term prognosis of patients with acute STEMI. Exp Ther Med. 2017;13:604–608. 28352337 10.3892/etm.2016.3987 PMC5348695

